# Supplementary material for: Complex‐centric proteome profiling by SEC‐SWATH‐MS
Source: Mol Syst Biol. 2019 Jan 14;15(1):e8438. doi: 10.15252/msb.20188438 (PMC6346213; doi:10.15252/msb.20188438)
Supplement: Supplementary file 8 — Dataset EV7 [file MSB-15-e8438-s008.zip › feature_plots_string/Q9Y399.pdf]

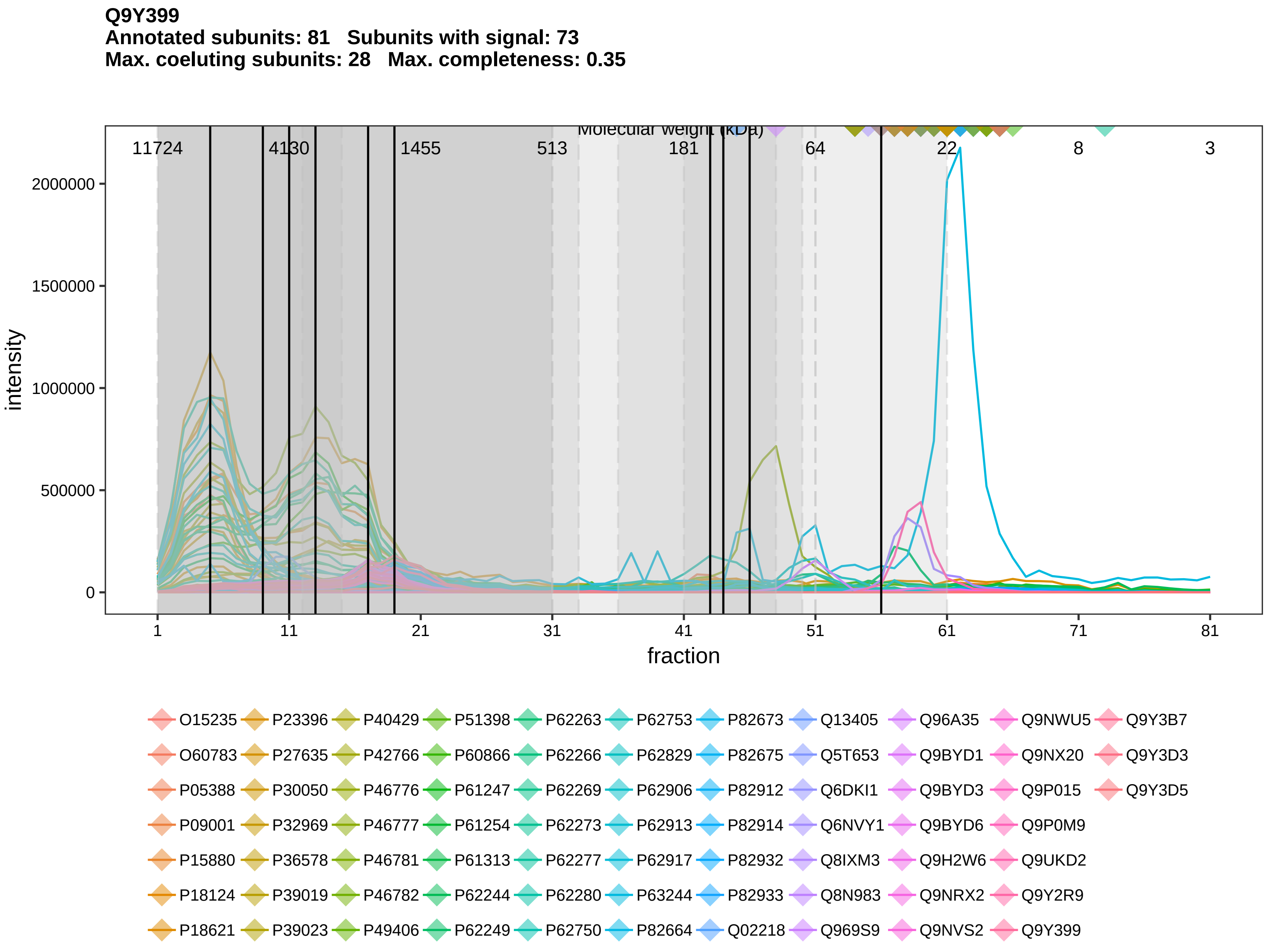

Q9Y399
Annotated subunits: 81 Subunits with signal: 73
Max. coeluting subunits: 28 Max. completeness: 0.35
Molecular weight (kDa)
11724
4130
1455
513
181
64
22
8
3
2000000
1500000
1000000
500000
0
intensity
1
11
21
31
41
51
61
71
81
fraction
O15235 P23396 P40429 P51398 P62263 P62753 P82673 Q13405 Q96A35 Q9NWU5 Q9Y3B7
O60783 P27635 P42766 P60866 P62266 P62829 P82675 Q5T653 Q9BYD1 Q9NX20 Q9Y3D3
P05388 P30050 P46776 P61247 P62269 P62906 P82912 Q6DKI1 Q9BYD3 Q9P015 Q9Y3D5
P09001 P32969 P46777 P61254 P62273 P62913 P82914 Q6NVY1 Q9BYD6 Q9P0M9
P15880 P36578 P46781 P61313 P62277 P62917 P82932 Q8IXM3 Q9H2W6 Q9UKD2
P18124 P39019 P46782 P62244 P62280 P63244 P82933 Q8N983 Q9NRX2 Q9Y2R9
P18621 P39023 P49406 P62249 P62750 P82664 Q02218 Q969S9 Q9NVS2 Q9Y399
